# Unveiling the Therapeutic Horizon: HPV Vaccines and Their Impact on Cutaneous Diseases—A Comprehensive Review

**DOI:** 10.3390/vaccines12030228

**Published:** 2024-02-23

**Authors:** Florica Șandru, Andreea-Maria Radu, Aida Petca, Mihai Cristian Dumitrașcu, Răzvan-Cosmin Petca, Alexandra-Maria Roman

**Affiliations:** 1 Department of Dermatovenerology, “Carol Davila” University of Medicine and Pharmacy, 020021 Bucharest, Romaniaandreea.radu@rez.umfcd.ro (A.-M.R.); alexandra-maria.roman@rez.umfcd.ro (A.-M.R.); 2 Dermatology Department, “Elias” University Emergency Hospital, 011461 Bucharest, Romania; 3 Department of Obstetrics and Gynecology, “Carol Davila” University of Medicine and Pharmacy, 020021 Bucharest, Romania; 4 Department of Obstetrics and Gynecology, Elias Emergency University Hospital, 011461 Bucharest, Romania; 5 Department of Obstetrics and Gynecology, University Emergency Hospital of Bucharest, 050098 Bucharest, Romania; 6 Department of Urology, ‘Carol Davila’ University of Medicine and Pharmacy, 020021 Bucharest, Romania; 7 Department of Urology, ‘Prof. Dr. Th. Burghele’ Clinical Hospital, 050659 Bucharest, Romania

**Keywords:** HPV vaccine, dermatology, HPV, warts, skin cancer, βHPV

## Abstract

Human papillomavirus (HPV) encompasses a diverse array of viruses, comprising approximately 200 serotypes that affect humans. While the majority of HPV strains are associated with benign skin or mucous membrane growths, a subset is implicated in severe health conditions, such as cervical, anal, vulvar, and vaginal cancers. Despite the established effectiveness of HPV vaccines in preventing cervical and anal carcinomas in particular, their therapeutic potential in addressing cutaneous diseases linked to diverse HPV strains remains an intriguing area of investigation. This narrative review critically examines the existing literature to assess the viability of HPV immunization as a therapeutic intervention for prevalent cutaneous conditions. These include genital and extragenital cutaneous warts, epidermodysplasia verruciformis, and keratinocyte carcinomas. The findings suggest a promising dual role for HPV vaccines in preventing and treating dermatologic conditions while emphasizing future research directions, including the immunization perspective against β-HPVs. Moreover, the presence of conflicting study outcomes underscores the imperative for larger-scale, randomized trials with well-matched control groups to validate the efficacy of HPV immunization in the dermatologic context. This review contributes valuable insights into the evolving landscape of HPV-vaccine applications in the field of dermatology.

## 1. Introduction

In the realm of virology, human papillomaviruses (HPVs) emerge as nonenveloped, double-stranded circular DNA viruses, infecting basal keratinocytes in both the skin and mucosal surfaces [1]. These viruses, comprising over 200 subtypes categorized into five major genera (alpha, beta, gamma, mu, and nu), exhibit significant DNA sequence diversity; the first three genera are the most prevalent ones [1,2].

Genital HPVs, mainly classified as α-HPVs and impacting mucosal surfaces, have been extensively studied due to their strong association with cancers, such as those affecting the cervix, anus, vulva, vagina, penis, and oropharynx. Research was conducted to combat the substantial disease burden, and efforts have led to the development of three highly effective HPV vaccines (the bivalent HPV vaccine (bHPV), the quadrivalent HPV vaccine (qHPV), and the nine-valent HPV vaccine (9vHPV)), specifically targeting genital α-HPV subtypes (HPV type 6, 11, 16, 18, 31, 33, 45, 52, and 58). However, these vaccines lack coverage for cutaneous HPV infections, particularly those caused by β-HPVs, which are more prevalent than anogenital infections and are highly correlated with skin malignancies [3].

Given the ubiquitous distribution of β-HPVs in the global population and their higher prevalence compared to α-HPVs, the predominant clinical manifestation of HPV is often cutaneous warts. Despite the diverse treatment options currently available, a considerable number of individuals grapple with persistent and refractory disease.

Recently, the highly recommended HPV vaccine has demonstrated its utility beyond its originally intended prophylactic purpose, being postulated as a treatment option for HPV-induced lesions, ranging from benign warts to dysplastic and neoplastic lesions [4,5,6,7,8]. Subsequently, our aim is to scrutinize the recent literature that has surfaced concerning the therapeutic applications of HPV vaccines in diverse cutaneous lesions.

Achieving complete resolution of the lesion should be the primary objective in treating all types of warts, rather than settling for a partial answer. Thus, our primary focus in analyzing the selected studies was on achieving absolute resolution during treatment.

## 2. Materials and Methods

The design of this study was that of a narrative review. We conducted methodical research on the PubMed database over a period of 5 years (from January 2018 to January 2024), using the key terms: “HPV vaccine” and “dermatology”, “warts,” and “condyloma”, respectively, obtaining a total 578 articles. The inclusion criteria encompassed original clinical studies involving human subjects presented in the English language, including case series and case reports. We excluded the papers that assessed both primary and secondary prophylactic efficacy of the HPV vaccine, review papers, and non-English papers.

We pursued three primary sections and included 30 studies: the potential of utilizing the HPV vaccine as a treatment for warts regardless of their location (21 studies), its application in addressing neoplasms (4 studies), and its effectiveness on HPV-related skin lesions in immunocompromised patients (5 studies). Of these, 15 were original studies, 5 were case series, and 8 were case reports. Our research comprises a total of 1437 patients, with 1405 being treated with the HPV vaccine for warts, 7 of them for neoplasia, and 25 of them for immunosuppression-related diseases.

The findings, along with the ensuing discussion points, have been organized into distinct categories, encompassing benign lesions like warts, cutaneous neoplasia, and the particularities of managing HPV lesions in immunosuppressed patients.

## 3. Results

### 3.1. Genital and Extragenital Warts

Reports have surfaced regarding the open-label utilization of human papillomavirus (HPV) vaccines as a therapeutic avenue for treating warts, thereby prompting further exploration within the research domain. To evaluate the treatment efficacy, the commonly employed system involved classifying the response as complete if there was a total clearance of lesions and partial if there was more than a 50% reduction in the number of lesions [9,10,11]. An illustrative instance is the case series described by Hayashi et al. in 2020, wherein the administration of the qHPV vaccine resulted in the complete resolution of multiple warts in two individuals at disparate ends of the age spectrum, namely 70 years old and 9 years old [12]. This observation was particularly noteworthy because it demonstrated the effectiveness of cross-linked immunity, as evidenced by the patients’ HPV genotyping indicating the presence of HPV 57 and 27, neither directly targeted by the qHPV vaccine [12]. Waldman et al. demonstrated more modest outcomes when assessing the qHPV vaccine as a therapeutic approach for cutaneous, extragenital warts in a cohort of 16 patients, two of whom were immunosuppressed. Only 44% of the patients achieved complete clearance of the disease, while 38% reported persistence or progression of the lesions [6]. In another study encompassing 30 patients with multiple extragenital warts, the evaluation of the qHPV vaccine administered at a three-dose regimen (at baseline, two months, and six months) intramuscularly in the triceps revealed a complete response rate of 46.67%. The majority of patients began to demonstrate clearance after the second injection [13]. Neither of these studies could identify any statistically significant differences regarding prior treatments, age, anatomic location, lesion number, and disease duration concerning treatment response. However, Kuan et al. reported a higher rate of complete response with a shorter median period of wart disease, although the difference was not statistically significant [11]. The aforementioned study, which investigated patients with recalcitrant acral warts undergoing qHPV vaccination, revealed a low complete response rate (30.8%) and a partial response rate (34.65%). Intriguingly, among nonresponders to the treatment, a discernibly lower median number of warts was observed in comparison to the responder groups [11]. Conversely, Choi et al. reported more favorable rates of genital-warts response following the administration of the qHPV vaccine (60%) during a mean follow-up period of 8.42 ± 3.27 months [14]. A more extensive randomized, controlled, partially blinded trial failed to establish a statistically significant therapeutic effect of the qHPV. However, consistent results were observed for both wart clearance at week 16 and the recurrence-free period (aOR (95%CI) = 1.46 (0.97 to 2.2) for the qHPV vaccine vs. placebo for being wart free at week 16 and remaining wart free between weeks 16 and 48 [15]. The study conducted by Kost et al. revealed a comparable lack of efficacy in both the qHPV and the 9vHPV vaccine. In all groups, comprising individuals vaccinated postdiagnosis of verruca vulgaris, those vaccinated prior to the diagnosis, and those unvaccinated, there was no discernible elevation in verruca vulgaris resolution rates (54.17% vs. 52.67% vs. 52.22%, *p* = 0.907) [16]. Moreover, upon stratification based on the vaccine type, therapeutic outcomes exhibited no significant variation, except for individuals receiving both vaccine types during the follow-up, wherein a notably lower verruca vulgaris complete resolution rate (25.00%, *p* = 0.006) was observed [16].

A corresponding study demonstrated the effectiveness of Gardasil9 in the therapeutic management of refractory genital warts in a cohort of five patients. After the patients underwent conventional local therapies (podophyllotoxin, imiquimod, cryotherapy, and CO_2_) for a mean duration of 2.6 ± 1.29 years, the administration of the third dose of Gardasil resulted in a reduction in the number of lesions: three instances of disease regression and two cases of complete remission. Additionally, within the same group, there was a notable enhancement in the patients’ reported quality of life, as measured by DLQI after vaccination (12.00 ± 2.12 vs. 5.90 ± 4.71, *p* = 0.0579) [17]. The effectiveness of the 9vHPV vaccine was established in a study involving 45 patients, where the average number of lesions was 15.7. Predominantly, the lesions were situated on the plantar site (57.8%), followed by periungual warts (44.4%) [18]. The overall complete response rate stood at 62.2%; however, a statistically significant difference surfaced when stratifying the patients by age group. Notably, the response rate was lower in patients aged over 26 years compared to those aged 9 to 26 years (55.0% vs. 84.0%, *p* = 0.049) [18]. A younger age (less than 45 years old) emerged as a notable factor, along with a smaller number of lesions (less than 10), a shorter duration of the disease (less than two years), and immunocompetence, collectively enhancing the clinical response to both qHPV and the 9vHPV vaccine, in conjunction with standard treatments (*p* > 0.05) [9]. Despite the inclusion of five immunosuppressed patients in the study, a statistically significant difference was evident in the cumulative complete and partial responses to treatments. Notably, 85% of patients subjected to combined therapy (standard therapy and HPV vaccination) exhibited a positive clinical response, whereas only 40% of patients undergoing standard treatments alone demonstrated a favorable outcome (*p* = 0.01) [9]. Interestingly, despite a subgroup of three patients having previously received the qHPV vaccine, the clinical outcomes did not show a statistically significant difference when compared to individuals exclusively administered the 9vHPV vaccine in the study (*p* > 0.05) [9]. Moreover, the 9vHPV vaccine demonstrated noteworthy outcomes in the treatment of young patients with both genital and extragenital warts following the administration of two doses [10,19]. Remarkably, a patient who received only one dose of the 9vHPV vaccine due to side effects demonstrated a significant reduction in the size of condylomas, with complete disappearance noted 1.5 years after the initial visit [20]. Nevertheless, a faster response was observed in a case described by Martin et al., wherein a 10-year-old girl achieved complete remission of a wart located on her finger just ten days after receiving the first dose of the bHPV vaccine [21].

The bHPV vaccine also demonstrated efficacy when administered intralesionally. In a study comparing intralesional and intramuscular administration of the bHPV vaccine, it was observed that 81.8% of patients achieved complete wart clearance with intralesional injection, compared to 63.3% with intramuscular injection (*p* = 0.287) [22]. However, the response rate was significantly faster in the intralesional group (*p* = 0.005) [22]. Additionally, it was noted that 80% of patients with distant warts in the intralesional group exhibited a complete response [22]. In contrast, a clinical trial with matched groups, comparing the intralesional administration of the bHPV vaccine to the topical application of 25% podophyllin cream, revealed that the Cervarix group required a significantly longer time to achieve complete remission (8.4 ± 1.6 vs. 2.3 ± 0.52, *p* = 0.001), despite necessitating substantially fewer treatment sessions (4.63 ± 0.66 vs. 6.82 ± 2.17, *p* = 0.0001) [23]. In the same study, the intralesional administration of the bHPV vaccine did not significantly outperform the topical application of 25% podophyllin cream twice weekly in achieving a complete disease response (45.5% vs. 27.3%, *p* = 0.21). Nevertheless, the topical podophyllin resin 25% exhibited a statistically significant higher rate of partial response in comparison to the HPV vaccine (*p* = 0.002), as patients treated with the HPV vaccine tended to either undergo complete remission or display no response at all [23].

Furthermore, a more recent study demonstrated that the qHPV vaccine’s intralesional administration achieved superior clearance rates for distant noninjected warts compared to the bHPV vaccine (87.5% vs. 66.5%) [24]. In comparing the efficacy between intralesional administration of qHPV and the bHPV vaccine, the clearance rate is significantly higher in the qHPV group (90% vs. 30%, *p* < 0.0001) [24]. However, it is noteworthy that, in both treatment groups, no wart recurrence was documented at the 6-month follow-up for the patients who initially responded [24]. Conversely, the efficacy of the intralesional bHPV vaccine was found to be similar to that of intralesional candida antigen injection (CR: 50% vs. 63.3%, *p* = 0.153), and both displayed superior effectiveness compared to cryotherapy (CR: 50% vs. 20%, *p* = 0.05, 63.3% vs. 20%, *p* = 0.001, respectively) [25]. Combination therapy, incorporating both candida antigen and HPV vaccination, has also been investigated. In a study conducted by Marei et al., it was revealed that the combination of intralesional candida antigen injection and intramuscular administration of the bHPV vaccine yielded a statistically significant and improved clinical response in patients with recalcitrant warts compared to candida antigen alone (CR: 70% vs. 40%, *p* = 0.014) [26]. This observation held true across various baseline characteristics, including age, sex, size, location, number, and duration of lesions [26]. Nevertheless, the intralesional administration of bHPV or qHPV vaccines in conjunction with candida antigen for anogenital warts (AGW) did not demonstrate superiority compared to intralesional candida antigen alone (*p* = 0.092) [27]. However, a noteworthy statistical difference was evident between the Gardasil 4/candida and Cervarix/candida groups (20% vs. 60%, *p* = 0.018). Nonetheless, the former is considered economically unfeasible [27]. Additionally, it was recently reported that the 9vHPV vaccine as an intralesional treatment led to 60% of patients achieving complete resolution of the disease. Intriguingly, unlike intramuscular vaccination, where a younger age was linked to a more favorable response, the intralesional administration showed that advanced age was associated with an improved score (*p*  <  0.05, β of −0.044 [confidence interval, −0.083 to −0.004]) for wart clearance [4] (Table 1).

### 3.2. HPV Vaccine as a Treatment in Cutaneous Neoplasia

The association between HPV 16 and cutaneous neoplasia affecting both the nail apparatus and the genital regions has prompted research into HPV vaccination as a potential alternative for managing these conditions. In this context, a case involving a 15-year-old girl diagnosed with Bowen’s disease affecting the nail folds of the thumbs, with confirmed HPV 16 positivity, was addressed through a combination of intramuscular administration of the Gardasil vaccine and cryosurgery. The reported outcome indicated complete remission, with no discernible signs of recurrence over six months [28]. Likewise, a case series involving three patients diagnosed with high-grade penile intraepithelial neoplasia (HPeIN) and confirmed HPV 16 positivity underwent treatment using the 9vHPV vaccine alongside topical imiquimod. Subsequent follow-up biopsies, conducted at intervals exceeding nine months, revealed no indications of persistent penile intraepithelial neoplasia. Additionally, immunostaining for HPV 16 yielded negative results [29]. An intriguing paper outlines the case of a 90-year-old woman presenting with multiple cutaneous basaloid squamous cell carcinomas (SCCs). In an innovative approach, the patient received the 9vHPV vaccine through both systemic (two doses) and intratumoral (four doses) administration. Notably, there was a discernible improvement in tumor size and a reduction in the number of lesions observed just two weeks after the initial two intratumoral administrations. Furthermore, 11 months following the initial intratumoral dose, no clinical or histological evidence of residual SCC was observed [30] (Table 2).

### 3.3. HPV Vaccine as a Treatment for Cutaneous Diseases in the Immunosuppressed Population

The efficacy of HPV vaccines extends to the treatment of immunocompromised populations, as evidenced by a case series demonstrating disease regression of skin warts following the standard three doses of the Gardasil9 vaccine. Notably, one of the patients had undergone multiple therapies over a span of 5 years without success in treating palmoplantar warts. However, this patient achieved nearly complete resolution just one month after receiving the third dose of Gardasil9. Furthermore, all five patients exhibited a statistically significant improvement in quality of life, as evidenced by the Dermatology Life Quality Index (DLQI) scores (11.4 ± 3.4 vs. 3.0 ± 4.5, *p* = 0.0114) [31]. Additionally, a retrospective study examined the therapeutic response following the initial dose of either the qHPV or 9vHPV vaccine in 18 patients diagnosed with palmoplantar warts. Among these individuals, 14 (78%) were immunocompromised, including 3 HIV-positive patients with CD4 counts between 100 and 200/mm^3^, one organ-transplant recipient, and four with common variable immune deficiency. Furthermore, the warts persisted for a median duration of 10 years, rendering most of them challenging to treat. At the twelve-month mark, two patients (11%) achieved complete remission (CR), while seven patients (39%) showed a partial response (PR) with a mean time to response of 3.5 months (ranging from 1 to 12 months). No significant factors were identified as associated with treatment response, including age, sex, immunocompromised status, smoking, wart location, presence of other HPV infections, duration of wart evolution, number of prior treatments, and the type or number of doses of vaccine received. Among responder patients, the recurrence rate was 44%, with a mean time to recurrence of 19 months. Additionally, in partial or nonresponder patients, delayed healing (either complete or partial) was observed in 44% of cases, with a mean time to recovery of 22 months [32] (Table 3).

## 4. Discussion

### 4.1. The Context of HPV Infection and Vaccination

Cutaneous lesions resulting from HPV exhibit a notably high prevalence within the general population, particularly among pediatric cohorts, immunocompromised patients, and sexually active individuals. In a 2020 cross-sectional study involving young adults, the prevalence of external genital lesions (EGL) was identified at 4.08%, with a higher occurrence in men (5.72%) compared to women (2.31%) (*p* < 0.001). The presence of genital lesions showed significant associations with male gender, infection by high-risk and multiple HPV types, a history of more than two sexual partners in the previous year, and the coexistence of other sexually transmitted diseases (STIs) [36]. The importance of sexual health is highly relevant in the context of HPV transmission, as emphasized by numerous studies. One such study indicates that individuals with a history of 16 or more lifetime sex partners had a significantly elevated risk of experiencing vaccine-preventable infections, showing a 13.4 times higher likelihood for men and a 14.7-fold increased risk for women, as opposed to those with two or fewer partners [37]. Nevertheless, the risk of acquiring HPV is influenced by various behaviors, including alcohol use, which has been linked to a heightened prevalence of EGL in women [36]. Moreover, former smokers, both men and women, exhibited a threefold increased risk of acquiring a specific vaccine-preventable HPV type compared to individuals who had never smoked [37].

The prophylactic effectiveness and safety of the HPV vaccine are clearly established in both men and women [38,39,40,41,42]. This was also demonstrated by a retrospective analysis involving 942,841 women aged 9–28. The study reaffirmed that the probability of a diagnosis of AGW was significantly lower in young women who were either fully or partially vaccinated (0.92% and 0.84%, respectively) compared to their unvaccinated counterparts (2.37%). Notably, for vaccinated women who were prescribed contraceptives before HPV vaccination, the protective effect of HPV vaccination was somewhat diminished. As expected, young women vaccinated with Gardasil^®^ or Gardasil9^®^ had a reduced probability of being diagnosed with AGW compared to their unvaccinated counterparts or those vaccinated with Cervarix^®^ [43]. Nonetheless, in a separate retrospective study encompassing 563,240 females, it was observed that a complete HPV4v vaccination schedule, which exhibited a 74% efficacy in reducing the incidence of genital warts, also resulted in an indirect protective effect on unvaccinated and HPV2v vaccinated girls [44].

While an ambispective observational study indicated a decline in the prevalence of HPV genotypes linked to most genital warts, which are targeted by the initial two prophylactic vaccines, there has been a rise in the occurrence of other HPV types. Moreover, there has been a slight rise in the occurrence of infections that involve multiple HPV genotypes or at least one high-risk HPV genotype [45].

While HPV vaccination has proven effective in reducing the onset of AGW and other HPV-related lesions, the same cannot be said regarding secondary prophylaxis. The administration of qHPV after the first AGW episode did not provide protection against a second AGW episode [46,47]. A similar observation was made in a study assessing the short-term recurrence of high-grade anal intraepithelial neoplasia (HGAIN) in HIV-positive men who have sex with men (MSM). In this study, no significant difference (*p* = 0.38) in cumulative HGAIN recurrence rates was observed between the group receiving three doses of qHPV (44/64, 68.8%) and the placebo group (38/62, 61.3%) [48].

Conversely, a significant decrease in the hospitalization rates for AGWs seems strongly linked to the widespread implementation of HPV vaccination programs. The notable reduction in hospitalizations is predominantly attributed to a substantial decline among females, dropping from 20.4 per 100,000 to 10.8 per 100,000. In contrast, males showed a marginal increase in hospitalization rates, although it did not reach statistical significance [49].

Furthermore, achieving herd immunity is paramount, although a protective effect, even in the presence of low vaccination rates, has been reported [50,51]. This trend is further exemplified by the fact that following the initiation of the publicly funded female-only qHPV vaccination program, the AGW incidence in Manitoba decreased by 75% for 16 18-year-old girls and 51% among unvaccinated boys [52].

In essence, achieving a swift reduction in HPV-related morbidity is attainable with extensive coverage of multicohort vaccination, but it is estimated that opting for vaccination solely among populations of a certain age would defer the control of HPV-related diseases by a minimum of a decade [53].

The qHPV vaccine is recommended as the primary prophylactic measure for AGW [54]. Achieving full vaccination schedules with either the qHPV or 9vHPV vaccines generates effective antibody responses, ensuring similar protection against external genital lesions, AGW, and cervical, vaginal, and vulval precancer lesions or cancer [36]. In a clinical trial evaluating both the qHPV and 9vHPV vaccines, it was found that, while the immunogenicity remains consistent, the effectiveness of protection varies among individuals with incomplete vaccination schedules. Single-dose assessments revealed a 53% reduction in the overall risk of testing positive for a specific vaccine-preventable HPV type in those who received the HPV vaccine compared to those given the active control (hepatitis A vaccine), a 65% reduction in the two-dose analysis and 100% reduction in the three-dose schedule [37]. One of the studies we examined provided support for the idea that the effectiveness of HPV vaccines is contingent upon the number of doses administered [11].

While the majority of studies reported the highest point estimates of efficacy with three doses, the variability in effectiveness based on the number of doses was reduced or eradicated in analyses stratified by age at vaccination, as younger patients exhibited comparable responses even with a lower number of doses [55]. However, concerning the safeguarding against infection from HPV 16 and 18, the two highly oncogenic genotypes, it has been demonstrated that a single-dose vaccination with either the bHPV, qHPV, or 9vHPVvaccines appears to be equally effective as the protection provided by two or three doses [55,56,57,58]. Despite the vaccine being protective for oneself, it does not display the same qualities regarding one’s partner. In a study of 167 couples, no evidence was found supporting the protection of HPV vaccination for partners of recently vaccinated recipients [37].

While it is reasonable to anticipate varying efficacies of the HPV vaccine in different populations, individuals living with HIV exhibit high antibody responses to both the bHPV and qHPV vaccines [59]. Additionally, age is a significant factor in this context. Vaccine-effectiveness estimates for younger adolescents aged 9–14 years ranged from approximately 74% to 93%, while for adolescents aged 15–18 years, the range was from 12% to 90%. These findings highlight that the HPV vaccine demonstrates its highest effectiveness against HPV-related disease outcomes when administered at younger ages, underscoring the crucial importance of timely vaccination [60]. Moreover, it was established in two of our included studies that vaccine effectiveness, when employed as a treatment, is also contingent on age [9,18].

Managing HPV is of the utmost importance, especially considering findings from a study by Lee et al. in South Korea. The results regarding health-related quality of life (HRQoL) suggest that in males with genital warts (GW) and females with HPV-related diseases (such as high-grade dysplasia requiring ablation treatment), there is a noticeable negative impact on well-being and HRQoL scores. The study highlights a more significant effect on female GW patients compared to those with other HPV-related diseases (cervical intraepithelial neoplasia). Previous research has indicated that patients with GW experience significantly lower quality of life (QoL) and a substantial psychosocial burden, mainly when the infection is symptomatic, visible, and located in the genital region, leading to increased social stigma [61].

### 4.2. General Perception of HPV Vaccination

The potential reduction in the burden of HPV-related diseases among young, HPV-unexposed adolescents through vaccination is evident; however, a substantial gap exists between the targeted rates for achieving robust herd immunity, advocated by public health groups, and the actual vaccination rates [62]. In a 2022 study conducted in Germany, awareness of HPV and its role as a cancer-causing agent was found to be significantly age-dependent, with younger individuals exhibiting higher awareness. Despite this, only 43.6% of 25- to 34-year-olds were familiar with HPV, and merely 26.7% within the same age group were aware of its role in cancer causation. Additionally, there was a notable gender difference, with German women showing significantly higher awareness of HPV and HPV vaccination (34.2% and 56.4%, respectively, age dependent) compared to men [63].

The absence of well-defined regulations regarding the prescription, administration, and delivery methods of vaccinations has allowed factors like insufficient knowledge, cultural beliefs, and personal attitudes to influence the vaccination scenario in Greece, Reunion Island, and Romania [64,65,66]. Inadequate information and skepticism towards vaccinations among parents and caregivers stand out as the primary contributors to the low coverage of HPV vaccinations.

Men who have sex with men (MSM) are identified as one of the groups facing a higher susceptibility to HPV and HIV infections. Studies indicate that MSM face an elevated risk of HPV infection and prolonged presence of the virus, harboring various HPV types and experiencing a swifter progression to HPV-related diseases leading to malignancies. In a 2022 study involving 902 men who have sex with men (MSM) aged 18 and older, the findings revealed that 83.0% of the participants were aware of condyloma acuminata (CA), while 56% had knowledge of the HPV vaccine. However, a substantial portion of MSM in the study demonstrated insufficient understanding of these topics, with 41.2% to 64.3% lacking knowledge regarding transmission routes, symptoms, risks, and treatments related to CA. Behavioral intentions, particularly vaccination intention, were strongly linked to factors such as having friends or family members who received the HPV vaccine or having discussions about HPV. Unfortunately, only 34.8% of the MSM in the study reported having friends or family members vaccinated. Notably, 85.1% of MSM from 31 regions in China expressed willingness to be vaccinated against CA [67].

Nevertheless, following the introduction of a publicly funded HPV vaccination program for gay, bisexual, and other men who have sex with men (GBM) aged 26 years or younger in Canada, significant improvements in HPV vaccine coverage among young GBM have been evident within just over five years of implementing the targeted program [68].

### 4.3. HPV Vaccination as a Therapeutic Outlet

The existing literature indicates a broadened application of HPV vaccination beyond its approved usage, serving as a therapeutic intervention for HPV-associated cutaneous and mucosal conditions. Research supports the utilization of the commercially available three-dose series qHPV vaccine for treating conditions like cutaneous warts, nonmelanoma skin cancers (NMSC), and recurrent respiratory papillomatosis (RRP). Additionally, several noncommercial HPV vaccines have shown clinical efficacy in addressing AGW, cervical intraepithelial neoplasia (CIN), anal intraepithelial neoplasia (AIN), and vulvar intraepithelial neoplasia (VIN) [7,69]. Broadly speaking, prophylactic vaccines typically work by preventing future infections through the production of antibodies by B cells. In contrast, the elimination of pre-existing viral infections is often achieved through T-cell-mediated immune responses. HPV vaccines consist of noninfectious particles that resemble HPV and originate from the L1 capsid protein, which is unique to each subtype of HPV. Notably, there is homology between the L1 capsid proteins found in HPV subtypes associated with common warts, such as HPV subtypes 1–4, and those present in HPV vaccines [4,10]. This similarity may potentially trigger cross-immunity, eliciting an immune response against a broad range of HPV subtypes, including those leading to AGW. Furthermore, HPV vaccines include adjuvants that enhance immune responses, as demonstrated by more robust vaccine antibody responses compared to those induced by natural infection [4,23,70]. These vaccines have been shown to upregulate levels of Th1 cytokines, including TNF-α and IL-2, along with proinflammatory cytokines, such as IL-1α, IL-1β, and IL-6 [27]. The production of IgG-neutralizing antibodies directed against HPV-L1 capsid is hypothesized to contribute to crossed protection [23]. Moreover, there are reports that HPV vaccines could offer cross-protection against HPV strains beyond the specific subtypes the administered vaccine targets [71,72]. The 9vHPV vaccine, incorporating antigens from the α-HPV genera, has the potential to elicit humoral immunity to β-HPV, given the shared expression of L1 and L2 capsid proteins [33].

#### 4.3.1. HPV Vaccine for Genital and Extragenital Warts

Nevertheless, in our chosen studies, the standard vaccination protocol has demonstrated effectiveness in treating both genital and extragenital warts [23,39,47,48,49,50].

Significantly, a particular study in our analysis employed a fourth dose of the qHPV vaccine administered at seven months in immunocompetent patients. Remarkably, this intervention led to a complete response, effectively resolving multiple warts in one of the patients [51]. Despite the availability of numerous treatment options for warts, patients frequently grapple with warts that do not respond to existing therapies. In most of the studies we reviewed, recalcitrant warts were defined as those that remained uneradicated after undergoing more than two distinct therapies or persisted for over a year [23,28,49,50,52,53].

The demonstrated efficacy and safety of intralesional vitamin D3 and immunotherapy using Candida, MMR, Tuberculin PPD, and BCG in individuals diagnosed with warts have paved the way for exploring the potential of intralesional HPV vaccine immunotherapy [54,55,56,57]. The use of intralesional immunotherapy, either alone or in combination with acitretin, along with the newly introduced needling-induced immunotherapy, has been proven valuable in treating warts resistant to conventional treatments [58,59,60,61]. Immunotherapy has proven effective in achieving the resolution of primary lesions and distant and adjacent noninjected lesions and reducing the recurrence rate [62,63]. In the studies we reviewed, patients typically received between 0.1–0.3 mL of bi-/quadri-/nonavalent HPV vaccine, administered at two-week intervals, with a maximum treatment duration of 5 or 6 weeks [40,64,65]. In two studies we examined, patients who had previously received a different type of HPV vaccine were additionally administered the 9vvaccine. Interestingly, this unintended form of excessive immunization did not result in improved clearance outcomes. Furthermore, in one of the studies, these patients exhibited significantly lower verruca vulgaris resolution (*p* = 0.006) [29,66]. An intriguing observation is that the bHPV vaccine demonstrated statistically significant greater efficacy on larger-sized warts [42].

#### 4.3.2. HPV Vaccine for Nonmelanoma Skin Cancers

In the United States, there is an annual occurrence of 34,800 HPV-related cancers, with 32,100 attributed to the types targeted by the 9vHPV vaccine (9vHPV) [73]. Although cutaneous neoplasia is not the most prevalent, its diverse presentations, including basaloid and warty subtypes of penile SCC and other keratinocyte types, present a challenge [74,75,76]. The beta genus is recognized for its heightened association with the development of NMSC [77,78]. The progression from persistent HPV infection to precancers and ultimately invasive cancer spans many years, suggesting that it might be too early to observe the effects of HPV vaccination on invasive cancers [73].

The HPV vaccine has been used for the immunoprevention of cancer and precancerous lesions, such as high-grade intraepithelial vulvar or anal lesions, and its effectiveness has been confirmed through numerous papers in the specialized literature [79,80,81,82]. In immunosuppressed patients, particularly those who are HIV positive, intraepithelial neoplasia can be concealed within condylomas, which highlights the need for intensive prophylactic programs [82]. However, it is important to note that the vaccines cannot prevent diseases related to HPV types that are not targeted by the vaccines [79,80,81].

Nevertheless, there are case reports and case series highlighting the potential utility of the HPV vaccine as a treatment modality for patients with such neoplasms. In an attempt to reduce the occurrence of cutaneous skin cancers, a male in his 70s and a female in her 80s, both diagnosed with SCC and basal cell carcinoma (BCC), received treatment involving the three-dose series of the qHPV vaccine. Before vaccination, the male patient had an annual average of 12 new SCCs and 2.25 new BCCs, while the female had a mean of 5.5 SCCs and 0.92 BCCs per year. After 16 months from the first dose, the male patient exhibited a reduction to 4.44 SCCs and 0 BCCs per year (a 62.5% decrease in SCCs and a 100% reduction in BCCs). In contrast, at a 13-month follow-up, the female patient experienced a decline to 1.84 SCCs and 0 BCCs per year (a 66.5% reduction in SCCs and a 100% reduction in BCCs) [7,83]. Remarkable outcomes were also observed in the three studies we identified during our search, specifically in relation to penile cancer, Bowen’s disease, and cutaneous basaloid SCCs [28,29,30].

#### 4.3.3. HPV Vaccine—A Therapeutic Option for Immunocompromised Patients

Within the immunosuppressed population, notably among HIV-infected patients and those undergoing long-term immunosuppressive therapy, the prevalence of HPV infection was found to be high, at 86.2% and 70.5%, respectively. The overall HPV infection rate in the immunosuppressed cohort reaches 75.6%. Moreover, a substantial proportion of these individuals, precisely 41.1%, experienced multiple HPV infections [84]. Data indicate that not only do HPV infections occur more frequently in these patients, but the course of the infection may also contribute to accelerated oncogenesis [85,86]. Thus, the consideration of pretransplant HPV vaccination becomes imperative to prevent anal high-grade intraepithelial lesions and cancer arising from anal high-risk HPV (hrHPV) infection in kidney-transplant recipients (KTRs) [87]. Among organ-transplant recipients, the most prevalent malignancy is SCC, a condition strongly linked to chronic HPV infection [88]. With the growing population of patients undergoing immunosuppressive therapy, it is advisable to broaden the recommendation for the use of the 9vHPV vaccine to encompass all immunocompromised individuals [89,90].

In men living with HIV, the prevalence of HPV infection and AGW is notably high, standing at 79% and 12%, respectively. The persistence rate reaches 35%, and it is notably higher among patients with a low CD4 count [91]. Nevertheless, individuals who are stable on antiretroviral treatment with a CD4 count above 350 and those not on treatment with a CD4 count above 500 exhibit strong responses to vaccines [92,93,94].

Immunosuppressed patients may experience therapeutic benefits from HPV vaccination, as demonstrated in a case report where complete clearance of recalcitrant warts occurred in a patient with idiopathic immune deficiency following qHPV vaccination, similar to one of the aforementioned studies [31,95]. Two of the selected studies also emphasized the efficacy of the HPV vaccine in severe manifestations of immunosuppression, such as giant condyloma acuminata and epidermodysplasia, resulting in complete remission of these otherwise challenging-to-manage cases [34,35].

Even in severe cases involving multiple keratinocyte carcinomas, where the β-HPV genera are associated with the development of SCCs and actinic keratoses, the intramuscular 9vHPV vaccine demonstrated significant effectiveness. Patient 1 witnessed an 88% reduction in new keratinocyte carcinomas per year, including an 87% decrease in SCCs and complete postinjection prevention of BCCs. Similarly, Patient 2 experienced a 63% reduction in the yearly incidence of keratinocyte carcinomas, with a 30% decrease in SCCs and complete prevention of BCCs [33].

### 4.4. HPV Vaccine Tolerability and Safety

As with many vaccines, the HPV vaccine has as its most common side effects localized reactions at the injection site, encompassing sensations of pain, redness, swelling, warmth, itching, induration, the formation of nodules, and urticaria. Systemic adverse effects are infrequent and include rare occurrences of seizure, hyperhidrosis, gout flares, and chills [96,97,98]. While the safety and efficacy of HPV vaccines have been established, reports of adverse effects in the form of autoimmune events, including Behçet’s disease, type 1 diabetes, lupus erythematosus panniculitis, Reynaud syndrome, etc., have been documented [99,100,101,102].

A widely discussed hypothesis suggests that vaccinations act as nonspecific activators of the immune response, potentially exacerbating pre-existing autoimmunity or developing de novo autoimmune diseases [103]. Another avenue through which autoimmunity may be induced is the molecular mimicry mechanism [100]. Moreover, there have been instances of the rare acquired autoimmune subepithelial bullous disease, linear immunoglobulin A bullous dermatosis (LABD), reported following qHPV vaccination [103,104]. Another hyperergic immune response presenting as Wells syndrome (eosinophilic cellulitis) was documented in two young patients, both of whom exhibited positive reactions to aluminum salt patch tests [105].

Furthermore, while our selected cases exhibited successful treatment of AEV, a distinct outcome was observed in a male patient with HIV. Post the second dose of the qHPV vaccine, his AEV significantly deteriorated, showing no signs of improvement despite local therapy, completion of the vaccine regimen, and antiretroviral treatment, which evoked a hypothesis suggesting a vaccine-induced occurrence analogous to immune reconstitution inflammatory syndrome (IRIS) [106].

The occurrence of pityriasis lichenoides (PL) has been associated as well with HPV vaccination, with a higher frequency noted in male patients. The potential mechanism involves cross-reactivity between protein molecules present in Gardasil 9^®^ and shared epitopes on keratinocytes. This interaction, coupled with the trauma from vaccination, could trigger PL [98]. Pityriasis rosea has been documented as an additional adverse reaction to HPV vaccination in the literature. It has also been exceedingly rare but sporadically reported following vaccinations against diphtheria, tuberculosis, poliomyelitis, and tetanus [107].

Moreover, an intriguing case report detailing an adverse reaction to HPV vaccination involves a 28-year-old female who, five years after completing the three-dose intramuscular schedule of Gardasil, developed cutaneous pseudolymphoma at the injection site [108].

The intralesional administration of either of the HPV vaccines was well-tolerated, with only transient pain and induration at the injection site and a low occurrence of erythema and contact dermatitis (36.4%) [4,23,24].

### 4.5. Future Direction

**Figure 1:**
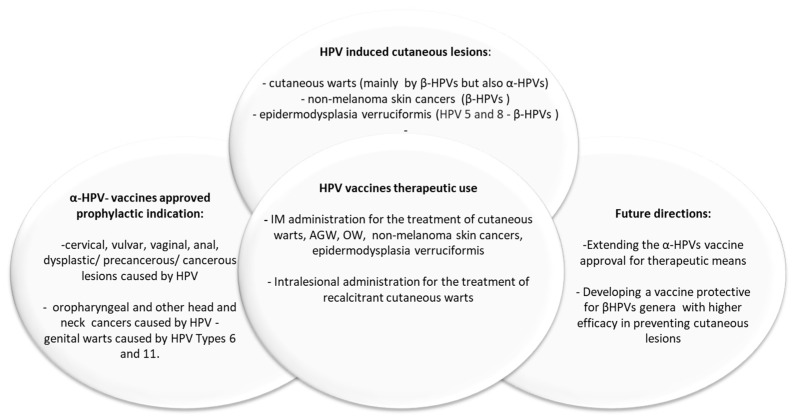
Indications and future directions for HPV vaccines.

As the efficacy of the currently available HPV vaccines remains inconsistent for skin HPV-related conditions, there have been various attempts to create novel vaccines targeting HPV genotypes that inhabit the skin (Figure 1).

The existing HPV vaccines, which utilize L1 VLPs, demonstrate strong immunogenicity and provide an effective defense against the specific HPV types they target. Nevertheless, they exhibit limited ability to guard against HPV types not covered by the vaccines [84,109,110,111]. Consequently, achieving comprehensive protection against all HPV-related cancers and warts necessitates incorporating VLPs from additional HPV types beyond those already included in the vaccines. As a substitute for L1 vaccines, targeting the L2 protein appears promising for developing a next-generation HPV vaccine. However, as previously stated, L2 does not create VLPs, resulting in lower immunogenicity. Fortunately, presenting peptides from the L2 protein on VLPs not only boosts the immunogenicity of the peptides but also enhances the vaccine’s potential to provide protection against a broader range of HPV types [112].

It is important to remark upon the differences between our current possibilities to prevent and treat α-HPVs and β-HPVs. α-HPVs are generally located in the mucosal tissues, and their high aggressivity made them the primary target for the currently available HPV vaccines. In contrast, there is limited efficacy of the current vaccines on β-HPV infections that cause cutaneous benign, precancerous, and cancerous lesions [113,114,115]. Hence, individuals who have received licensed HPV vaccines may still be susceptible to infection by β-HPV and the subsequent development of cutaneous lesions.

Initial preclinical studies on L1-based VLP vaccines show promise but are hindered by type-specific antibodies. Alternative approaches, such as L2-based and non-L2 chimeric VLP vaccines, demonstrate potential for broader coverage. Despite challenges, including lower neutralizing titers, efforts to enhance L2 immunogenicity through various strategies and exploration of DNA vaccine feasibility contribute to the evolving landscape of β-HPV prevention [115].

The development of the two-antigen vaccine, L2.RG2-VLP, signifies a noteworthy advancement in vaccine research. This vaccine demonstrates efficacy in animal models against known oncogenic αHPVs and a commendable focus on diverse βHPVs. The vaccine achieves this by integrating 20 amino acid-conserved protective epitopes derived from the minor capsid protein into the HPV16 and HPV18 L1 virus-like particles (VLP). This strategic incorporation enhances its potential utility and effectiveness in addressing a broad spectrum of HPV infections, particularly those associated with the diverse βHPV subgroup [116].

In principle, the presently accessible vaccines may demonstrate efficacy in mitigating cutaneous HPV infections to a certain extent. Nevertheless, the development of novel vaccines specifically designed to target the prevalent βHPV subtypes encountered in the skin is anticipated to yield more substantial contributions to both the prevention and treatment of such lesions. It is noteworthy that, until such innovative options become accessible, the existing vaccines may serve as a viable recourse for the management of particularly challenging cases.

## 5. Conclusions

HPV-related pathologies pose a significant healthcare burden, leading to diseases that are socially and psychologically taxing and often challenging to manage. While it is established that HPV vaccination can effectively decrease the prevalence and mortality of related diseases in the general population, its therapeutic application is still in the early stages of accumulating sufficient data. Despite the limited size of our reviewed study groups, which led to conflicting results, we assert that HPV vaccination constitutes a safe and effective therapeutic intervention for various HPV-induced diseases. These encompass conditions ranging from extragenital recalcitrant warts to keratinocytic skin cancers and epidermodysplasia verruciformis. Importantly, our findings suggest that this therapeutic approach remains efficacious, even in immunosuppressed individuals. However, to accurately assess the applicability and cost effectiveness of this approach, more randomized controlled trials involving diverse demographics and populations are needed to standardize the therapeutic use of HPV vaccines.

## Figures and Tables

**Table 1:** Treatment of genital and extragenital warts with HPV vaccines.

Author, Year of Publication	Type of Study	Population	Pathology	Vaccine Type	Results
Nofal, 2023 [24]	Parallel randomized controlled three-armed study	N = 50 patients with multiple recalcitrant warts N1 = 20 intralesional qHPV vaccine, mean age 31.7 ± 9.15 N2 = 20 intralesional bHPV vaccine, mean age 35.2 ± 10.32 N3 = 10 saline control, mean age 32.7 ± 6.68	Recalcitrant warts	Intralesional qHPV vaccine Intralesional bHPV vaccine	N1′ = 18 (90%) patients complete response N1″ = 2 (10%) patients partial response N1‴ = 0 patients no response N2′ = 6 (30%) patients complete response N2″ = 4 (20%) patients partial N2‴ = 10 (50%) patients no response N3′ = 10 (100%) no response N1 vs. N2 vs. Placebo: *p* < 0.0001
Fawzy, 2023 [27]	Randomized controlled trial	N = 80 patients N1 = 20 patients treated with intralesional Candida antigen only, mean age 41.8 ± 11.9 N2 = 20 patients treated with intralesional bHPV vaccine and Candida antigen N3 = 20 patients treated with intralesional qHPV vaccine and Candida antigen N3 = 20 patients treated with intralesional saline	Multiple AGW	Intralesional qHPV vaccine Intralesional bHPV vaccine	N1′ = 8 (40.0%) complete response N1″ = 8 (40.0%) partial response N1‴ = 4 (20.0%) no response N1 vs. N4: *p* < 0.001 N2′ = 4 (20.0%) complete response N2″ = 12 (60.0%) partial response N2‴ = 4 (20.0%) no response N2 vs. N4: *p* < 0.001 N3′ = 12 (60.0%) complete response N3″ = 4 (20.0%) partial response N3‴ = 4 (20.0%) no response N3 vs. N4: *p* < 0.001 N4′ = 0 (0.0%) complete response N4″ = 2 (10.0%) partial response N4‴ = 18 (90.0%) no response N2 vs. N3: *p* = 0.018
Bar-Ilan, 2023 [4]	Retrospective case series	N = 20 patients N1 = 13 adults N2 = seven children	Recalcitrant cutaneous warts	Intralesional 9vHPV vaccine	N′ = 3 poor response (0–24% improvement) and moderate response (25–49%, improvement); N″ = 5 (25%) excellent response (75–99% improvement); N″″ = 12 (60%) complete response with PGA score = 0
Ciccarese, 2023 [9]	Retrospective Study	N = 29 patients, average age of 39 years N1 = 14 patients received HPV vaccine simultaneously with standard treatments, average age of 39 years N2 = 15 patients with standard treatments, average age of 39 years	Recalcitrant agws/ows	9vHPV vaccine (IM)	N1′ = 9 (64%) complete response N1″ = 3 (21%) partial response N1‴ = 2 (15%) no response N2′ = 4 (27%) complete response N2″ = 2 (13%) partial response N2‴ = 9 (60%) no response *p* = 0.01
Nofal, 2022 [23]	Clinical trial	N = 44 immunocompetent patients N1 = 22 patients treated with intralesional bHPV vaccine, mean age 35.95 ± 10.63 N2 = 22 patients treated with topical podophyllin 25%, mean age 33.6 ± 8.1	Multiple AGW	Intralesional bHPV vaccine	N1′ = 10 (45.5%) complete response N1″ = 4 (18.1) partial response N1‴ = 8 (36.4%) no response N2′ = 6 (27.3%) complete response N2″ = 14 (63.6%) partial response N2‴ = 2 (9.1%) no response
Kost, 2022 [16]	Retrospective study	N = 336 patients with VV N1 = 48 patients vaccinated during follow-up, mean age 11.33 ± 2.91 N2 = 131 vaccinated before diagnosis, mean age 15.03 ± 2.55 N3′ = 157 unvaccinated patients, 11.94 ± 3.57	Verruca Vulgaris	9vHPV vaccine (IM)	N1′ = 9 (18.75%) complete response N1″ = 13 (27.08 %) partial response N1‴ = 26 (54.17%) no response *p* = 0.859 N2′ = 22 (16.79%) complete response N2″ = 40 (30.53%) partial response N2‴ = 69 (52.67%) no response *p* = 0.846 N3′ = 23 (14.65%) complete response N3″ = 52 (33.12%) partial response N3‴ = 82 (52.22%) no response
Nassar, 2021 [25]	Prospective study	N = 105 patients with multiple common warts N1 = 30 patients received 0.2 mL of intralesional Candida antigen, mean age 30.33 ± 17.88 N2 = 30 patients received 0.2 mL of intralesional bHPV vaccine, mean age 29.96 ± 18.85 N3 = 30 patients underwent cryotherapy, mean age 31.73 ± 17.80 N4 = 15 patients received 0.2 mL of intralesional saline, mean age 31.93 ± 17.58	Multiple common warts	Intralesional bHPV vaccine	N1′ = 19 (63.3%) complete response N1″ = 3 (10%) partial response N1‴ = 8 (26.7%) no response N2′ = 15 (50 %) complete response N2″ = 9 (30%) partial response N2‴ = 6 (20%) no response N3′ = 6 (20%) complete response N3″ = 13 (43.3%) partial response N3‴ = 11 (36.7%) no response N4′ = 0 (0%) complete response N4″ = 1 (6.7%) partial response N4‴ = 14 (93.3%) no response *p* < 0.001
Shin, 2022 [18]	Open-label, uncontrolled, single-arm study	N = 45 patients with multiple recalcitrant warts, mean age 28.7 ± 15.5 years	Multiple recalcitrant warts	9vHPV vaccine (IM)	N1′ = 28 (62.2%) complete response N1″ = 4 (8.9%) partial response N1‴ = 13 (28.9%) no response
Couselo-Rodriguez, 2021 [19]	Case report	N = 1 female, 11-year-old patient with genital warts	GW	9vHPV vaccine (IM)	Partial response—a pedunculated lesion in the anterior commissure of the labia majora persisted
Marei A, 2020 [26]	Prospective study	N = 40 patients, mean age 32.5 ± 24.7 years N1 = 20 patients received intralesional Candida antigen injection alone, mean age 31 ± 12.9 years N2 = 20 patients received combined treatment of bHPV recombinant HPV vaccine and intralesional Candida antigen, mean age 29 ± 8.47 years	Recalcitrant warts	Intralesional bHPV vaccine (IM)	N1′ = 8 (40%) complete clearance N1″ = 5 (25%) partial response N1‴ = 7 patients (35%) showed no response N2′ = 14 (70%) complete clearance in a combined therapy group N2″ = 4 patients (20%) showed partial response N2″ = 2 patients (10%) showed no response *p* = 0.014 statistically significant in favor of the combination therapy group
Nofal, 2020 [22]		N = 44 adult patients N1 = 22 patients received intralesional bHPV vaccine, mean age 29.27 ± 8.7 years N2 = 22 patients were injected with intramuscular bHPV vaccine, mean age 30.27 ± 12.2 years	Multiple recalcitrant common warts	bHPV vaccine (IM)	N1′ = 18 (81.8%) complete response N1″ = 2 partial response N1‴ = 2 no response. N2′ = 14 (63.3%) complete response N2″ = 6 partial response N2‴ = 2 no response
Hayashi, 2020 [12]	Case series	N = 3 immunocompetent male patients	Multiple warts—HPV typing: HPVs 57, 27	qHPV vaccine (IM)	N′ = 2 complete responses after the 3rd or 4th dose N″ = 1 no response
Gilson, 2020 [15]	Randomized, controlled, multicenter, partially blinded factorial trial.	N = 503 patients with AGW, mean age = 31 years; N1 = 125 received IMIQ plus qHPV, mean age 31 ± 10 N2 = 126 received PDX plus qHPV, mean age 31 ± 10 N3 = 126 received IMIQ plus placebo, mean age 32 ± 10 N4 = 126 received PDX plus placebo, mean age 30 ± 10 N’ = 12 (2.4%) participants were HIV positive	AGW	qHPV vaccine (IM)	aOR (95% CI) = 1.46 (0.97 to 2.20) for qHPV vaccine versus placebo for being wart free at week 16 and remaining wart free between weeks 16 and 48
Kuan, 2020 [11]	Retrospective study	N = 26 patients received HPV qHPV vaccine as an adjunctive treatment	Recalcitrant acral warts	qHPV vaccine (IM)	N1′ = 8 (30.8%), complete response, median age (range) = 27.5 (14–44) N1″ = 9 (34.65%) partial response, median age (range) = 36 (8–77) years N1‴ = 9 (34.65%) less than 50% improvement in their warts, median age (range) = 38 (21–64),
Bossart, 2020 [17]	Case series	N = 5 male patients without comorbidities with recalcitrant genital warts		9vHPV vaccine (IM)	N′ = 2 complete remissions N″ = 3 disease regressions
Kost, 2020 [10]	Case report	N = 9-year-old female patient	Multiple verruca vulgaris	9vHPV vaccine (IM)	Complete response, no recurrence at 9 months
Waldman, 2019 [6]	Retrospective study	N = 16 patients N′ = 2 immunosuppressed patients	Extragenital recalcitrant warts	qHPV vaccine (IM)	N′ = 7 (44%) complete clearance, N″ = 6 (38%) persistent or new warts N‴ = 3 (19%) lost to follow-up after HPV vaccination
Yang, 2019 [13]	Retrospective study	N = 30 patients, mean age = 21.43 ± 11.86	Multiple warts	qHPV vaccine (IM)	N′ = 14 (46.67%) complete response N″ = 5 (16.67%) partial response N‴ = 11 (36.67%) no response
Choi, 2019 [14]	Prospective study	N = 26 patients N1 = 16 underwent surgical excision, mean age 35.8 ± 11.2 years N2 = 10 vaccinated, mean age 26.1 ± 6.0 years	GW	qHPV vaccine (IM)	N′ = (60%) complete response
Kazlouskaya, 2019 [20]	Case report	N = 1 female, 79-year-old	Giant condyloma acuminata—HPV16, HPV18, HPV6, HPV11 Genotypes	1 dose of 9vHPV vaccine (IM)	Complete response after only one dose of the 9vHPV vaccine
Martin, 2018 [21]	Case report	N = 1 female, 10-year-old with a common wart	Cutaneous refractory wart	bHPV vaccine	Complete response

Intramuscular (IM), oral warts (OWs).

**Table 2:** HPV vaccine as a treatment for cutaneous neoplasia.

Authors, Year of Publication	Type of Study	Studied Population	Treatment	Results
Kim 2021 [29]	Case series	One male, 70-year-old with high-grade PEIN showing HPV 16 integration	9vHPV vaccine	Repeat biopsies at 13 months and 34 months showed no evidence of PEIN; Clearance of HPV 16 on repeat immunostaining
		One male, 46-year-old with high-grade PEIN showing HPV 16 integration		Repeat biopsies 11 months later showed no remaining PEIN; HPV 16 immunostaining was negative
		One male, 67-year-old with PEIN showing HPV 16 integration		Repeat biopsy 9 months later showed no remaining PEIN; HPV 16 immunostaining was negative
Jeon 2020 [28]	Case report	Fifteen-year-old girl with verrucous plaque involving the nail folds of the thumbs With HPV 16 positivity	Cryosurgery and qHPV vaccine	No sign of recurrence for 6 months
Nichols 2018 [30]	Case report	Ninety-year-old woman with multiple cutaneous basaloid SCCs	9vHPV vaccine systemically and intratumorally	Clinical improvement was observed 2 weeks after the second intratumoral dose of the 9vHPV vaccine with a reduction in tumor size and number. Eleven months after the first intratumoral dose, there was no clinical or histologic evidence of residual SCC.

Penile intraepithelial neoplasia (PEIN), scuamocellular carcinoma (SCC).

**Table 3:** HPV vaccine as a treatment for cutaneous diseases in the immunosuppressed population.

Authors, Year of Publication	Type of Study	Studied Population	Treatment	Results
Nichols 2022 [33]	Case series	N = 2 male patients with multiple prior kcs N1 = 1 patient with liver transplant N2 = 1 patient with Chron’s disease	9vHPV vaccine	N1′ = 88% reduction in kcs/year N2′ = 63% reduction in kcs/year
Merio, 2022 [32]	Retrospective study	N = 18 patients with palmoplantar warts, median age = 39.5 years	qHPV or 9vHPV	N1 = 2 patients (11%) in complete remission N2 = 7 patients (39%) in partial remission
Namuduri 2020 [34]	Case report	Forty-six-year-old woman with giant condyloma acuminatum of the vulva and AIDS	CO_2_ laser ablation + qHPV vaccine + acitetrin	After 2 years of treatment—complete remission
Bossart 2020 [31]	Case series	N = 5 immunosuppressed patients with recalcitrant skin warts	9vHPV vaccine	N1 = 1 patients with complete regression N2 = 4 patients with disease regression
Maor 2018 [35]	Case report	Fifty-year-old woman with renal transplant and acquired epidermodysplasia verruciformis	qHPV vaccine	One month after completion of the three doses of the qHPV vaccine the lesions were flattened

## Data Availability

Data sharing is not applicable.
